# Supplementary figures and images for: Role of hepatocyte RIPK1 in maintaining liver homeostasis during metabolic challenges
Source: eLife. 2025 Jan 31;13:RP96798. doi: 10.7554/eLife.96798 (PMC11785375; doi:10.7554/eLife.96798)

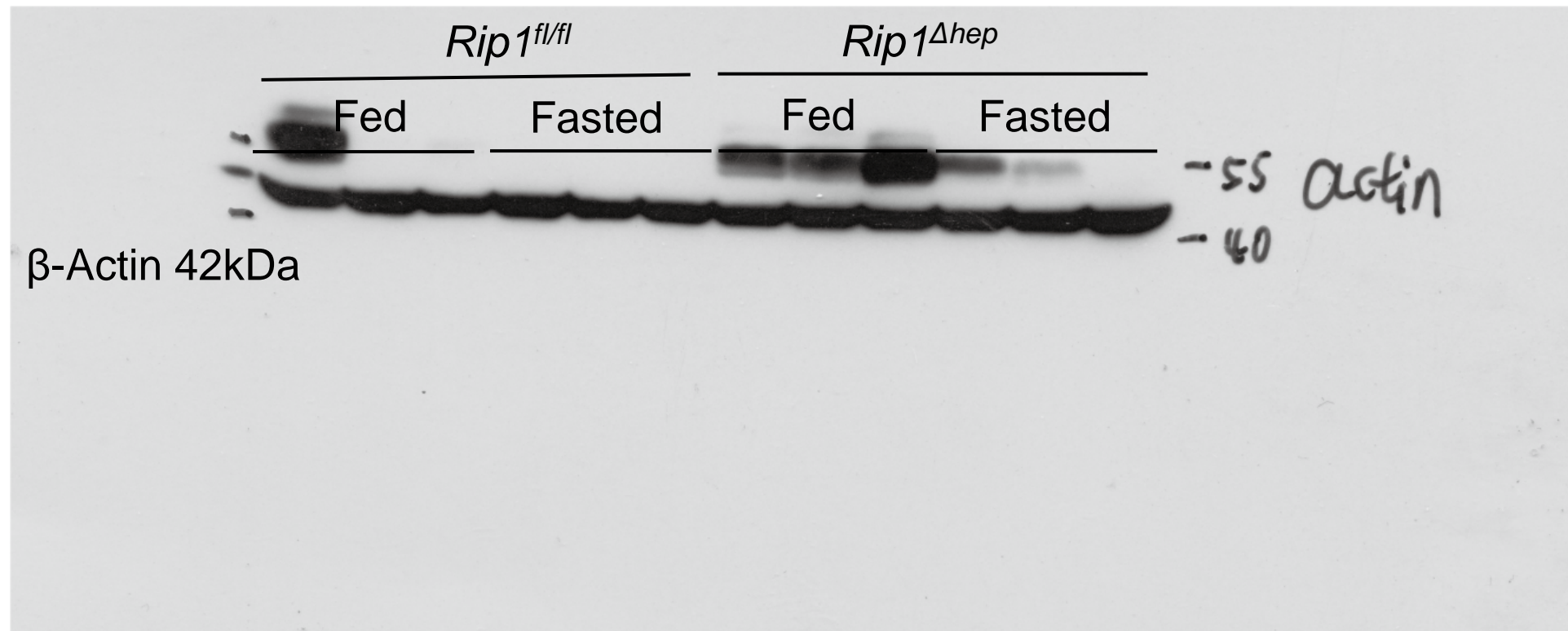

170-  
130-

*Rip1<sup>fl/fl</sup>*

*Rip1<sup>Δhep</sup>*

Fed

Fasted

Fed

Fasted

65-

70-

-170

-130 RI

-100

-70

RIPK1 78kDa

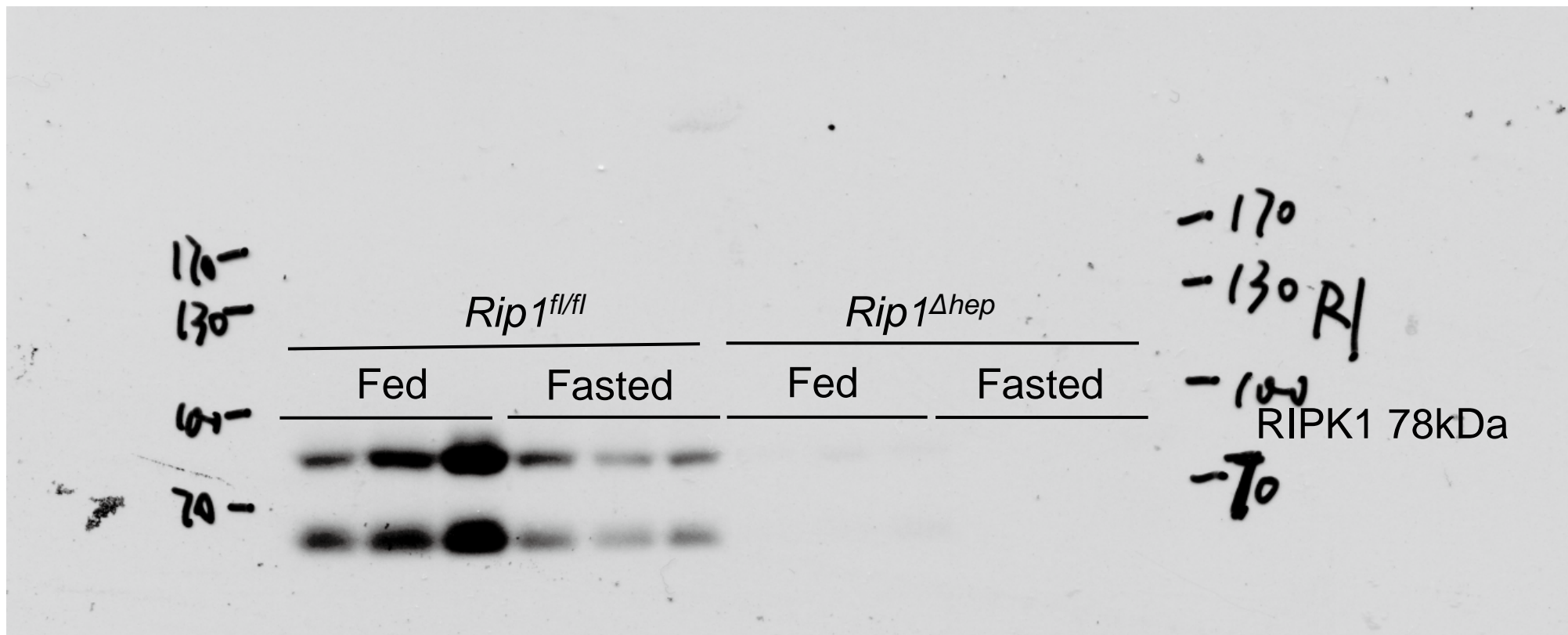

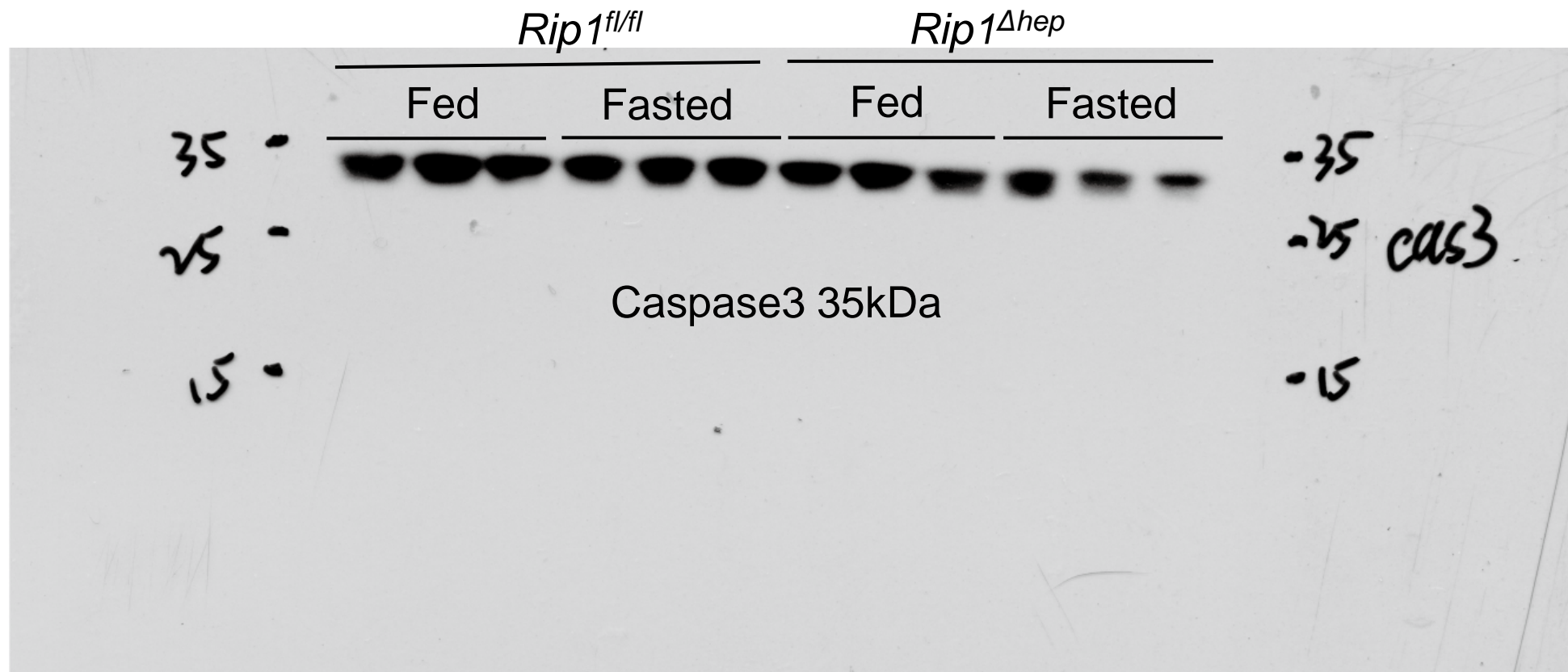

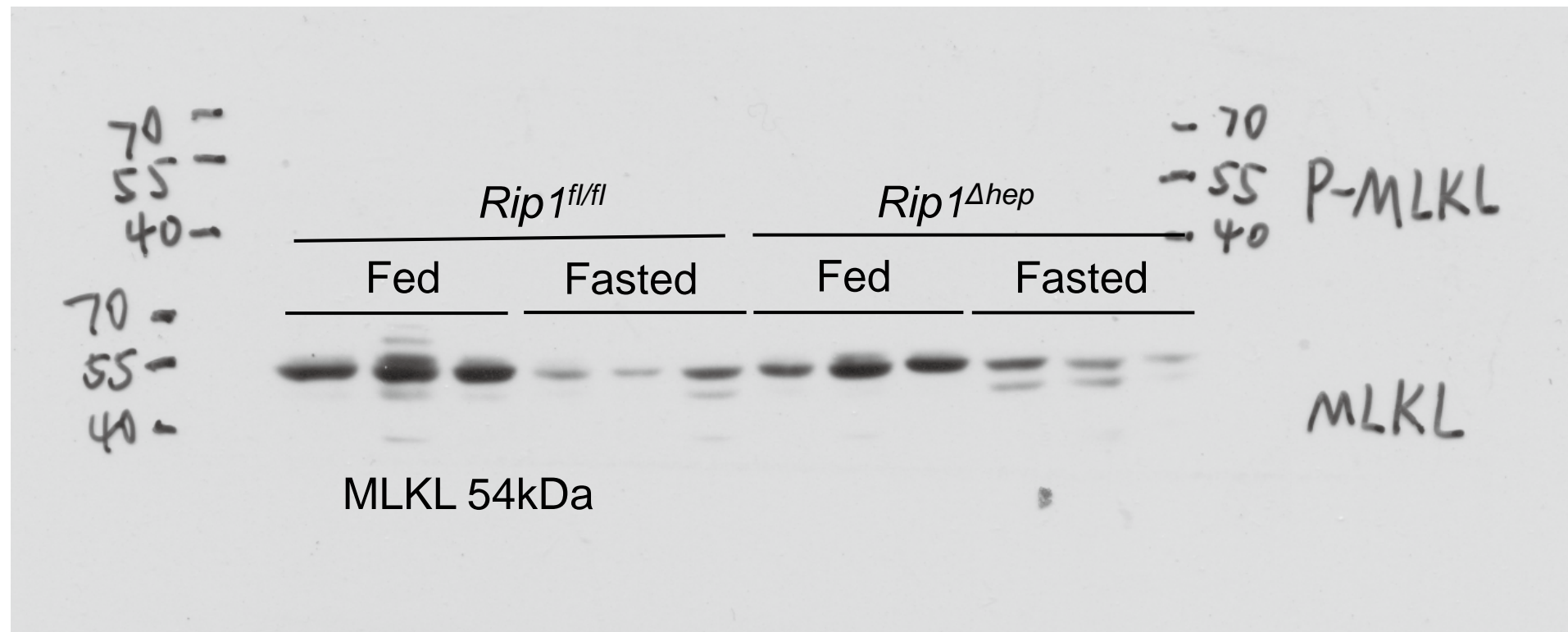

Supplement: Figure 1—figure supplement 1—source data 1. [file elife-96798-fig1-figsupp1-data1.pdf]

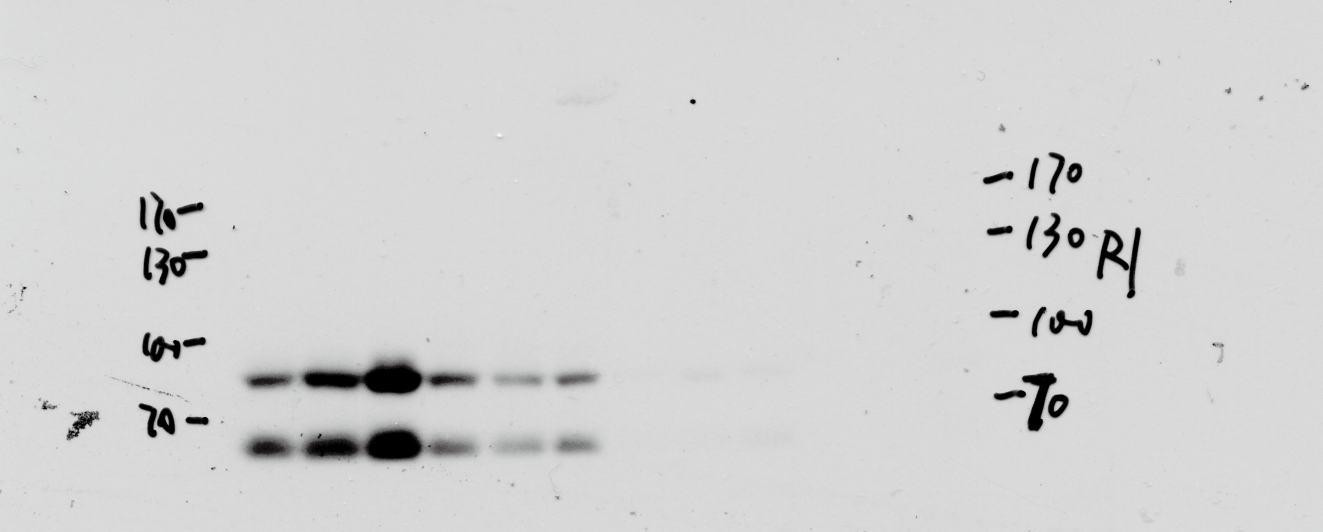

Supplement: Figure 1—figure supplement 1—source data 2. [file elife-96798-fig1-figsupp1-data2.zip › Figure 1-figure supplement 1-source data2/RIPK1.tif]

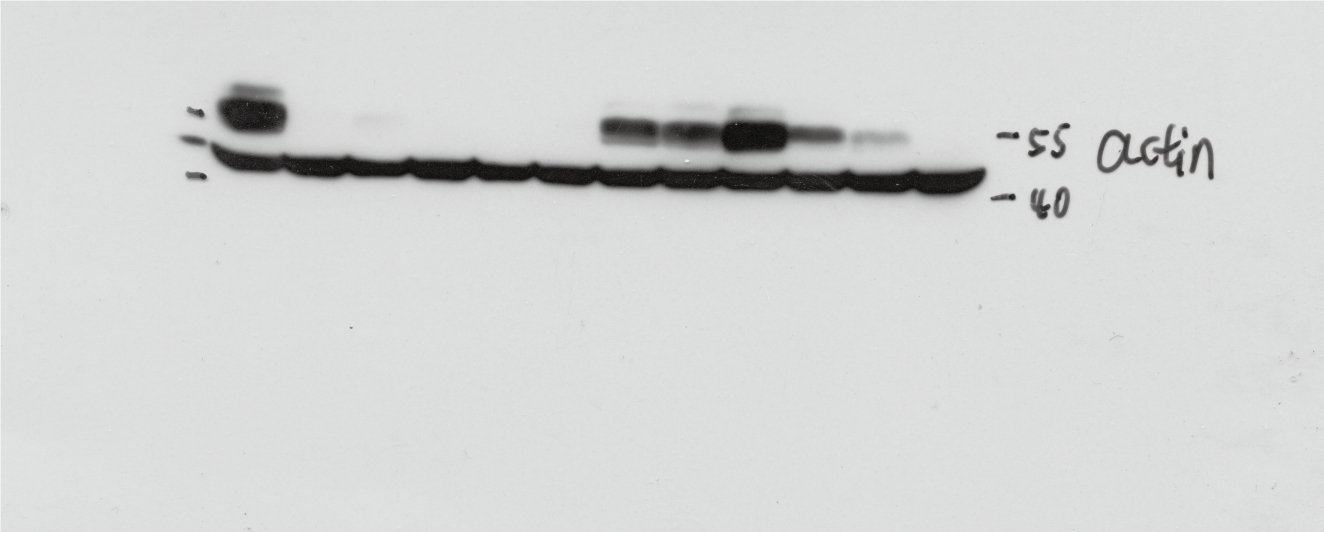

Supplement: Figure 1—figure supplement 1—source data 2. [file elife-96798-fig1-figsupp1-data2.zip › Figure 1-figure supplement 1-source data2/actin.tif]

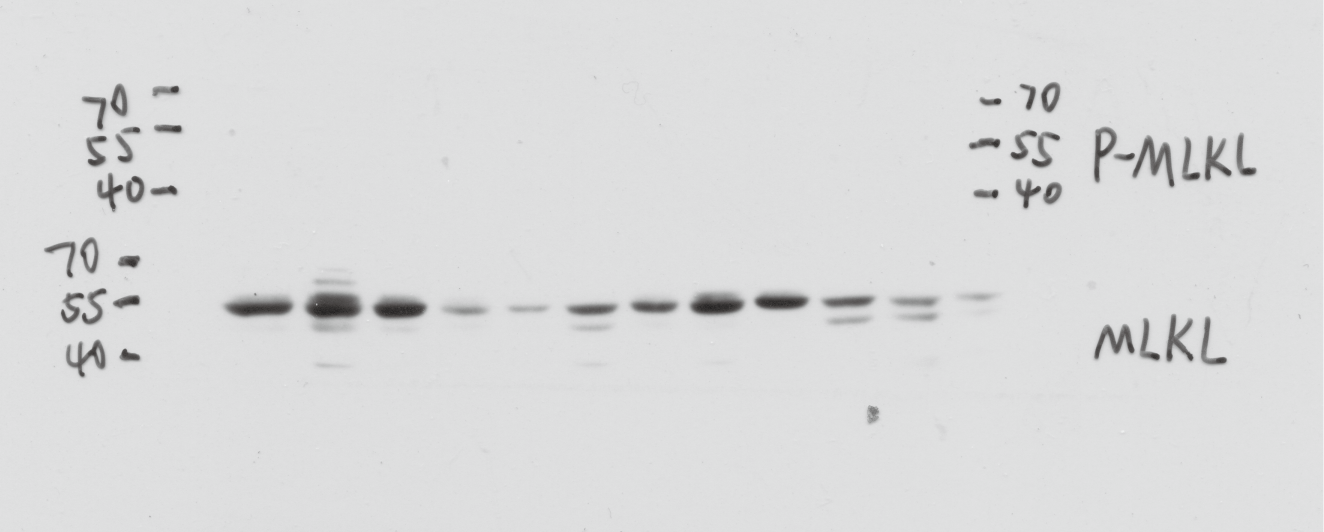

Supplement: Figure 1—figure supplement 1—source data 2. [file elife-96798-fig1-figsupp1-data2.zip › Figure 1-figure supplement 1-source data2/MLKL.tif]

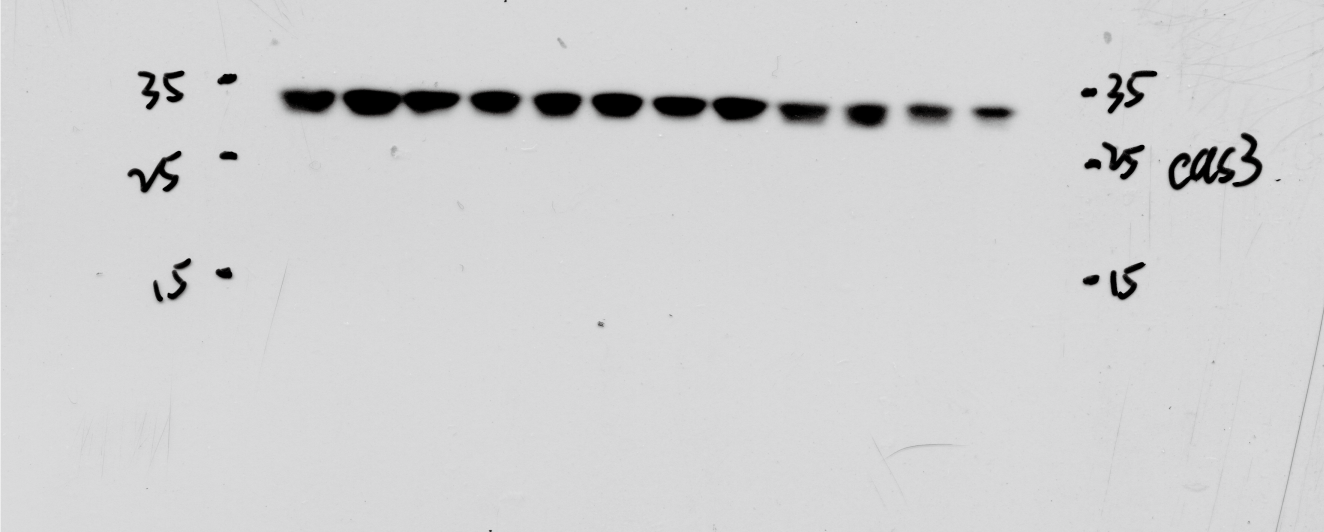

Supplement: Figure 1—figure supplement 1—source data 2. [file elife-96798-fig1-figsupp1-data2.zip › Figure 1-figure supplement 1-source data2/CAS3.tif]

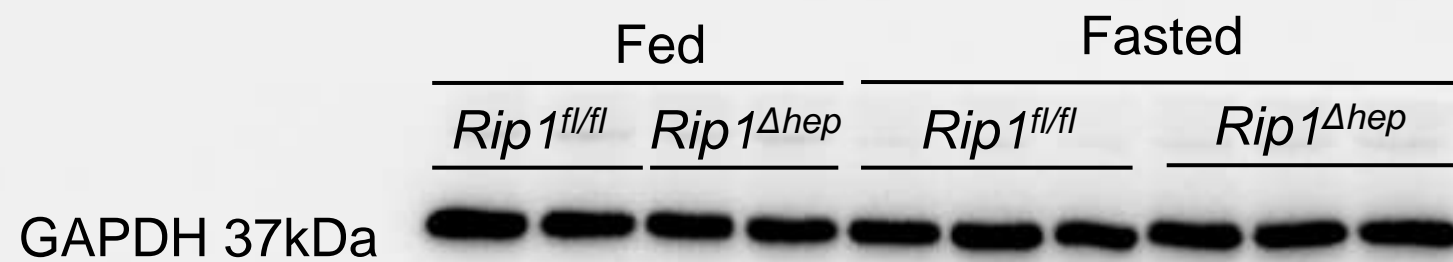

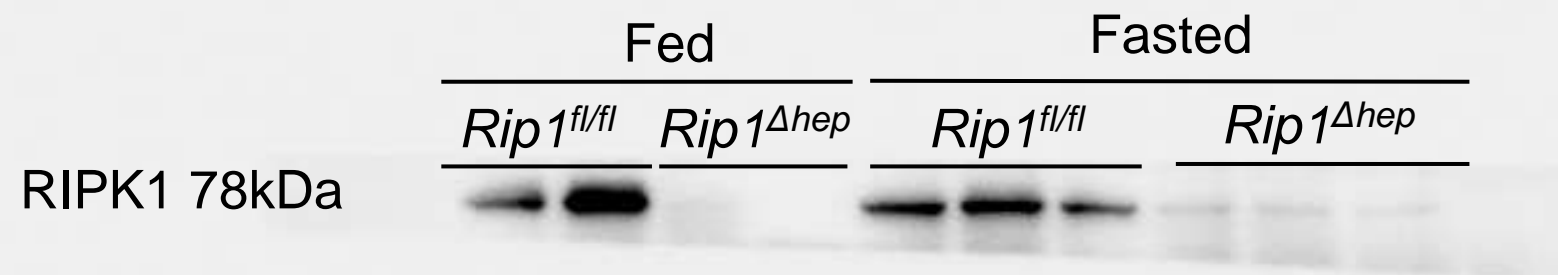

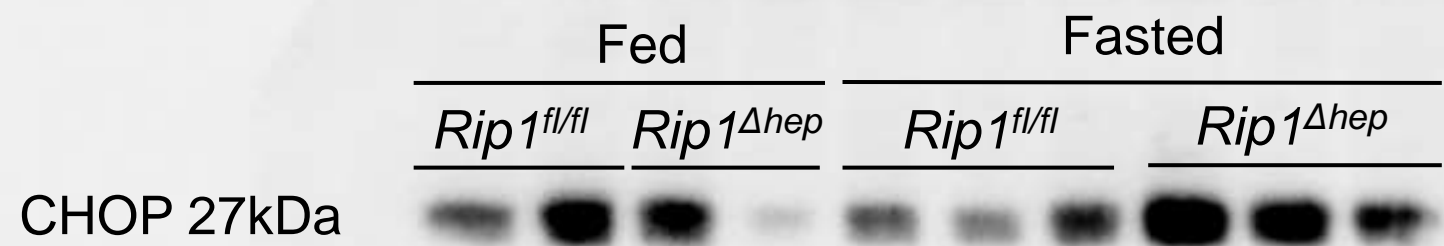

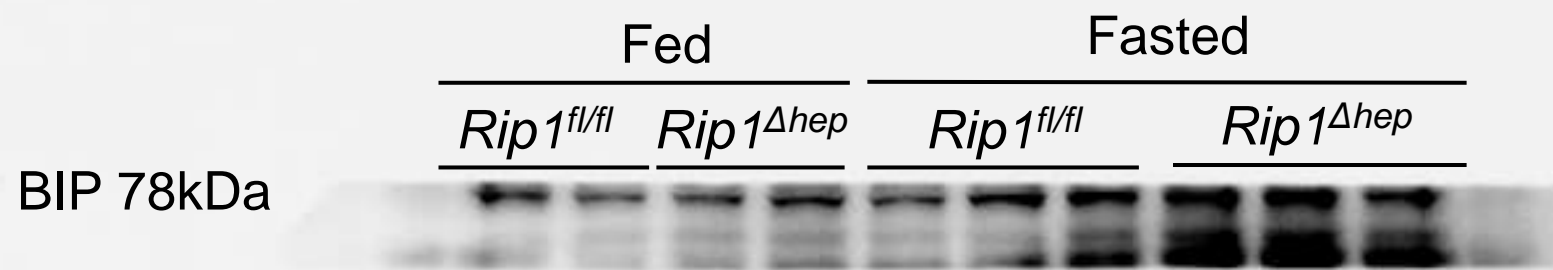

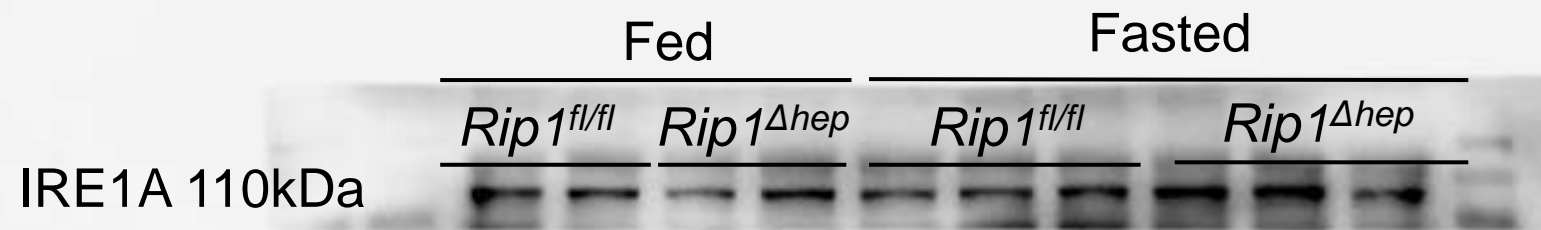

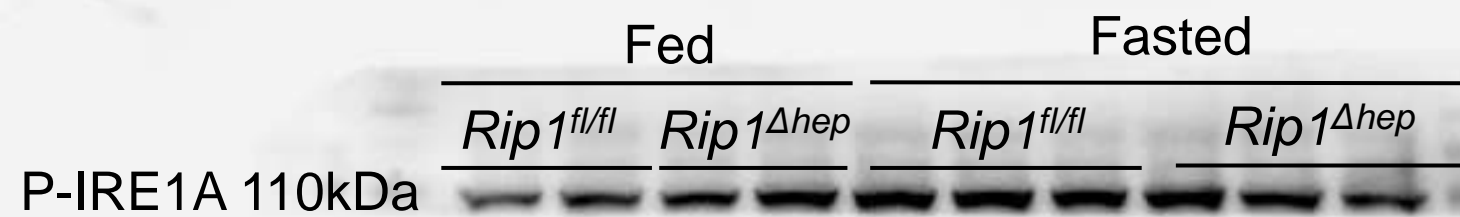

Supplement: Figure 5—source data 1. [file elife-96798-fig5-data1.pdf]

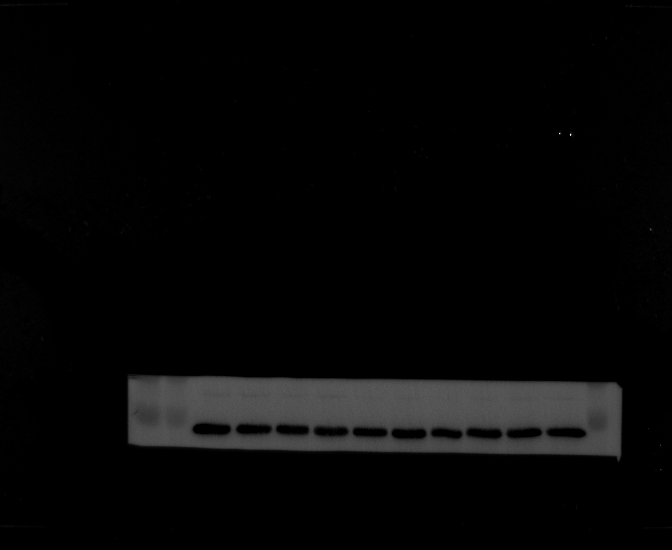

Supplement: Figure 5—source data 2. [file elife-96798-fig5-data2.zip › Figure5-source data2/GAPDH_marker.tif]

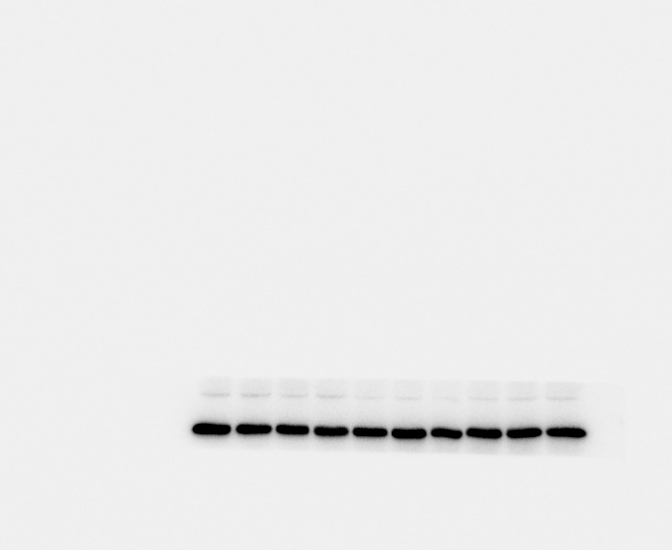

Supplement: Figure 5—source data 2. [file elife-96798-fig5-data2.zip › Figure5-source data2/GAPDH_sample.tif]

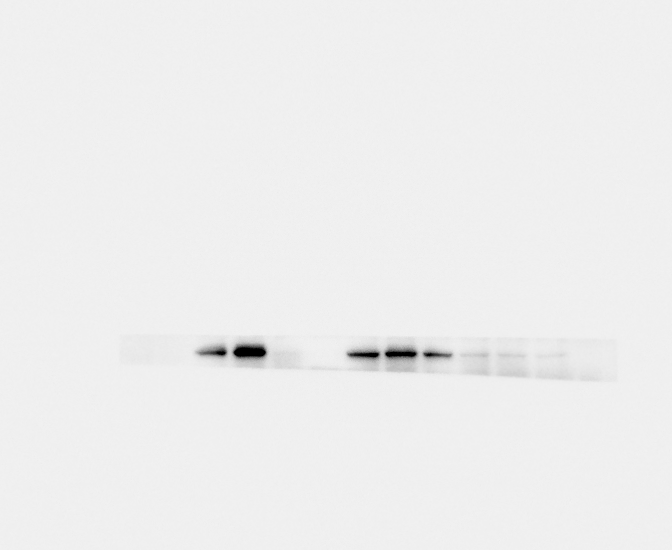

Supplement: Figure 5—source data 2. [file elife-96798-fig5-data2.zip › Figure5-source data2/RIP1_sample.tif]

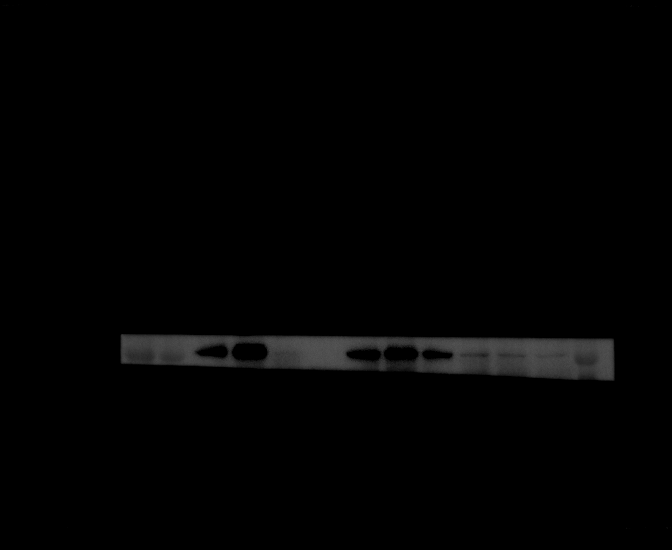

Supplement: Figure 5—source data 2. [file elife-96798-fig5-data2.zip › Figure5-source data2/RIP1_marker.tif]

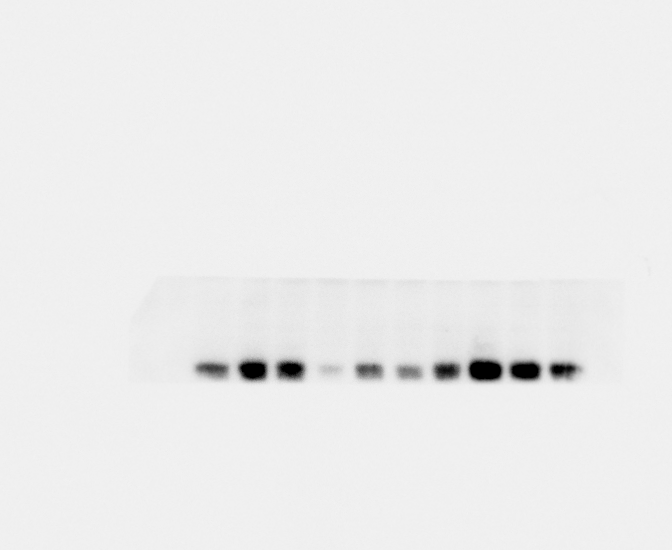

Supplement: Figure 5—source data 2. [file elife-96798-fig5-data2.zip › Figure5-source data2/CHOP_sample.tif]

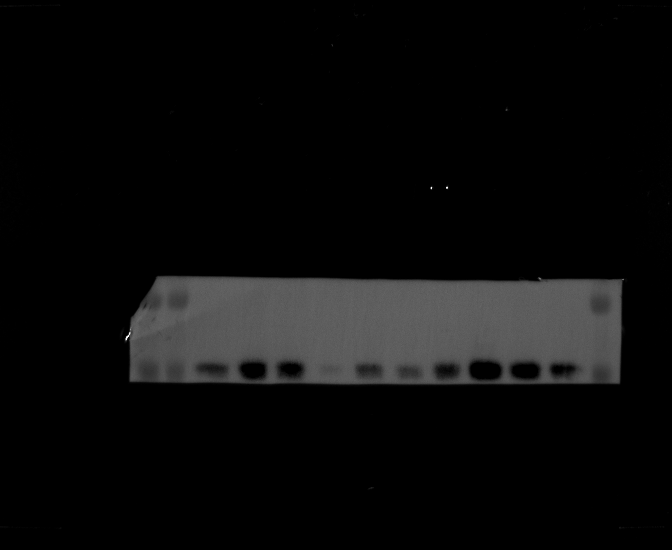

Supplement: Figure 5—source data 2. [file elife-96798-fig5-data2.zip › Figure5-source data2/CHOP_marker.tif]

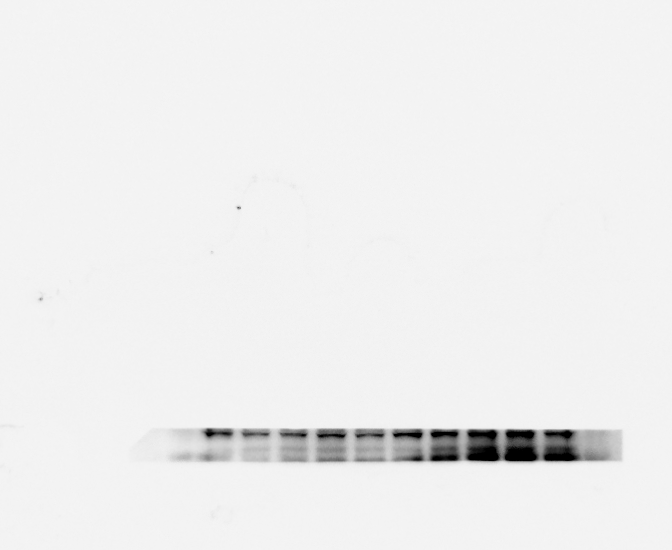

Supplement: Figure 5—source data 2. [file elife-96798-fig5-data2.zip › Figure5-source data2/BIP_sample.tif]

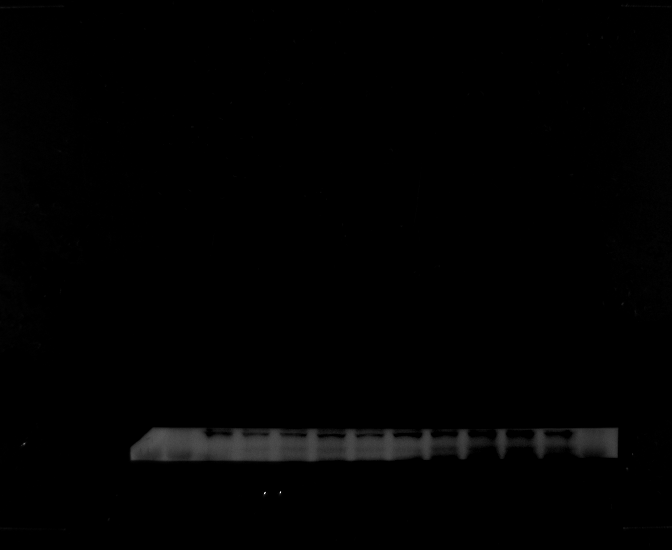

Supplement: Figure 5—source data 2. [file elife-96798-fig5-data2.zip › Figure5-source data2/BIP_marker.tif]

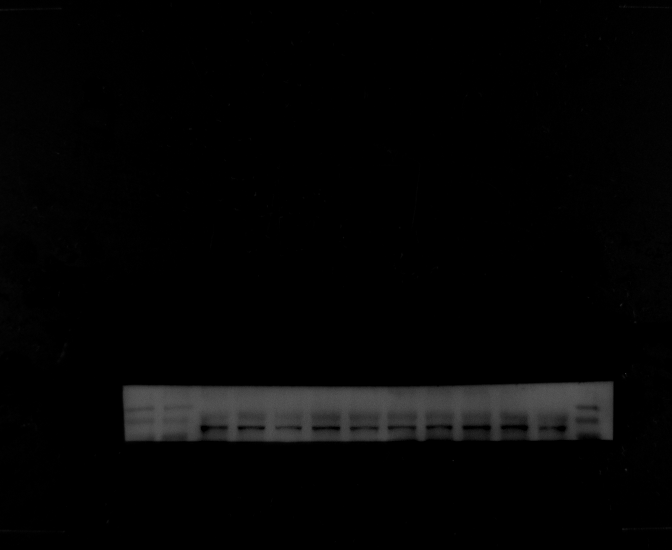

Supplement: Figure 5—source data 2. [file elife-96798-fig5-data2.zip › Figure5-source data2/IRE1A_marker.tif]

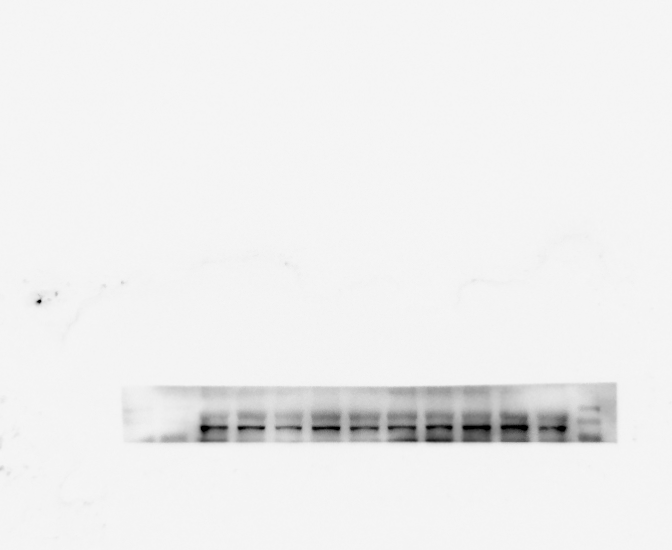

Supplement: Figure 5—source data 2. [file elife-96798-fig5-data2.zip › Figure5-source data2/IRE1A_sample.tif]

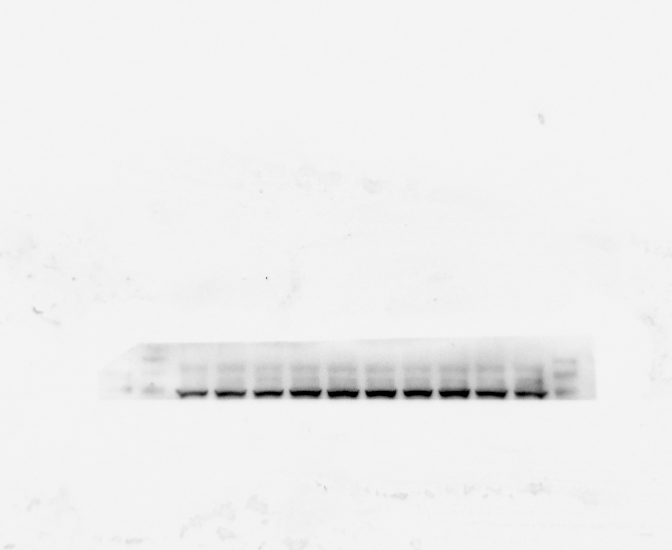

Supplement: Figure 5—source data 2. [file elife-96798-fig5-data2.zip › Figure5-source data2/P-IRE1A_sample.tif]

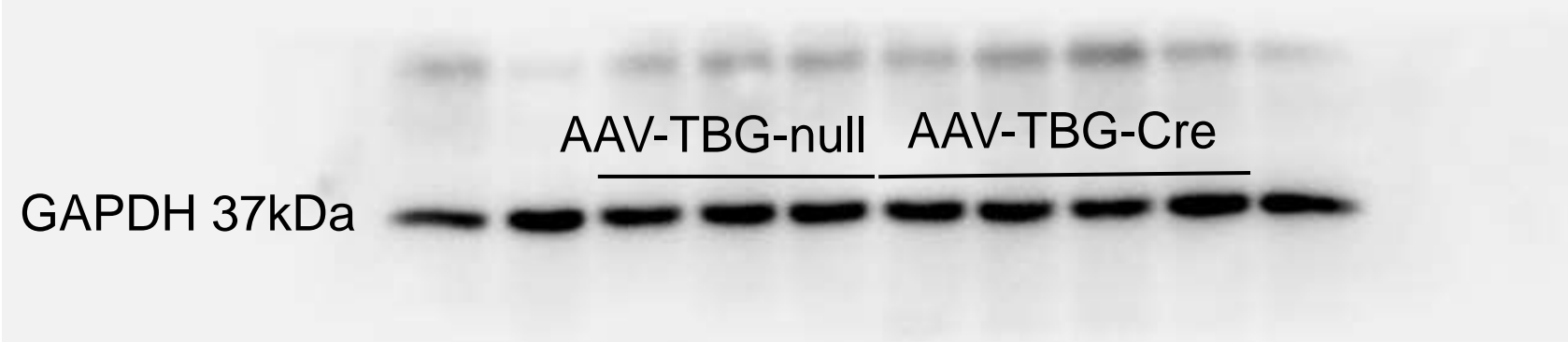

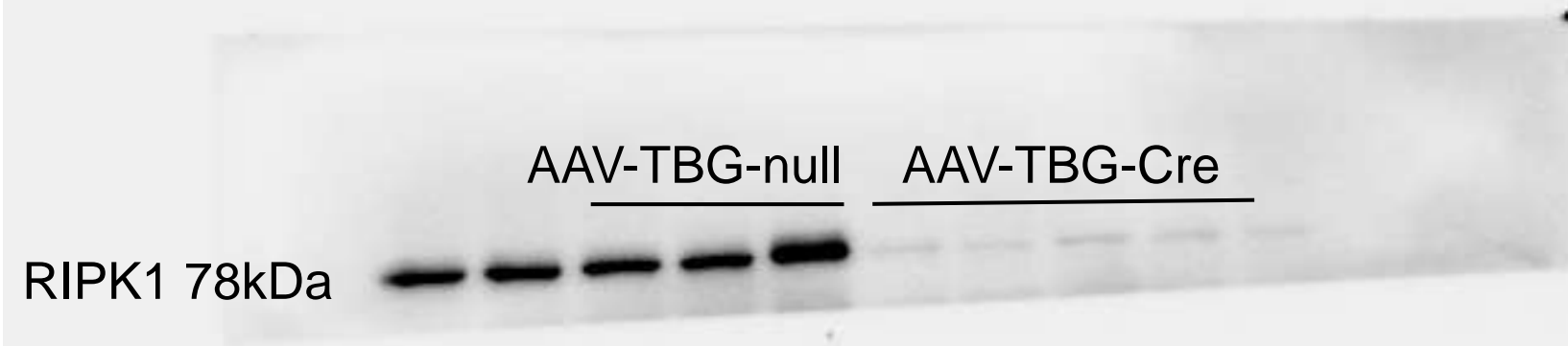

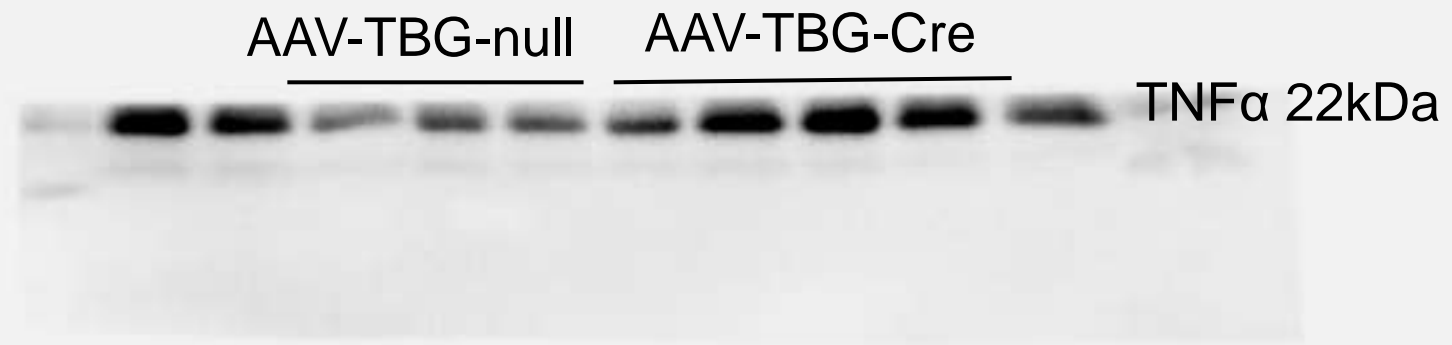

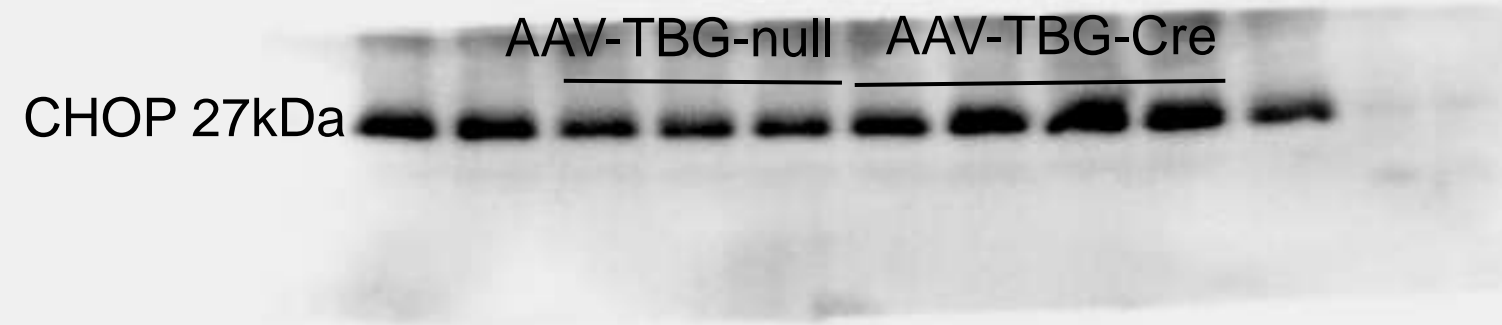

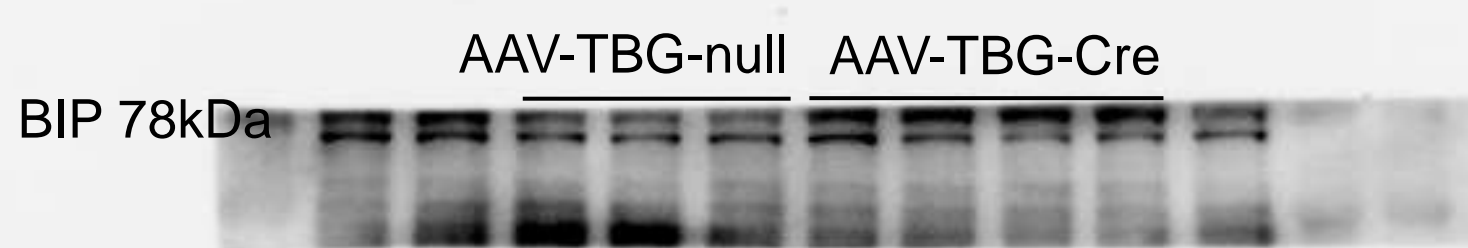

IRE1 $\alpha$  110kDa

AAV-TBG-null

AAV-TBG-Cre

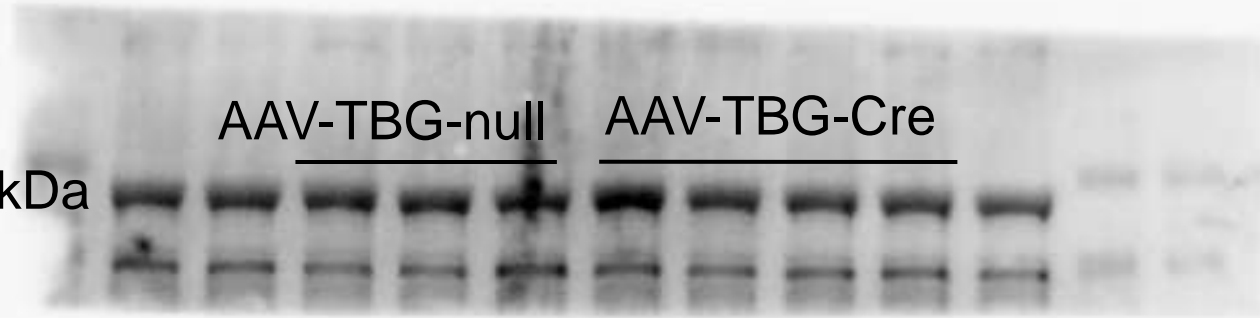

pIRE1 $\alpha$  110kDa

AAV-TBG-null

AAV-TBG-Cre

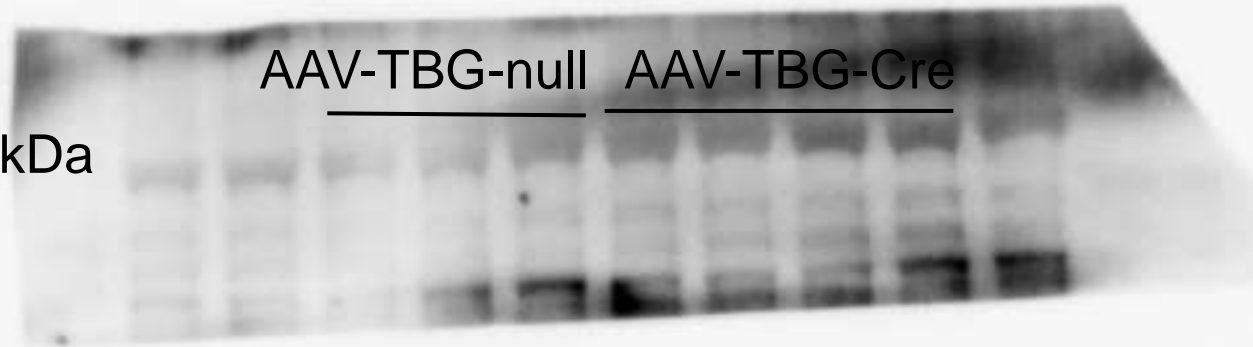

Supplement: Figure 6—source data 1. [file elife-96798-fig6-data1.pdf]

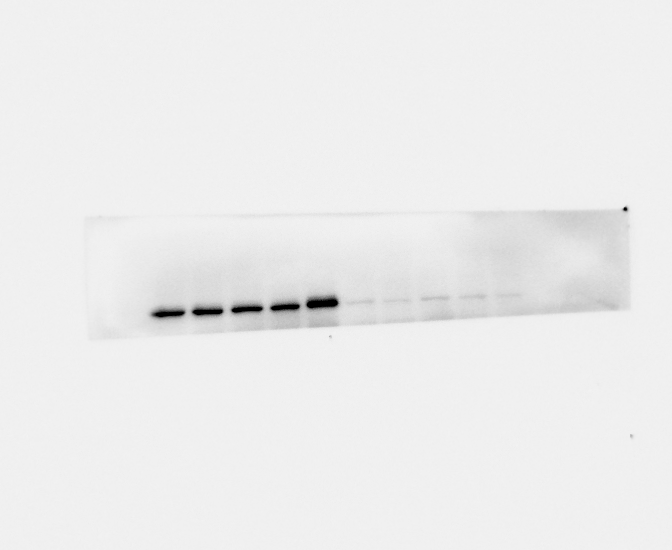

Supplement: Figure 6—source data 2. [file elife-96798-fig6-data2.zip › Figure6-source data2/RIP1_sample.tif]

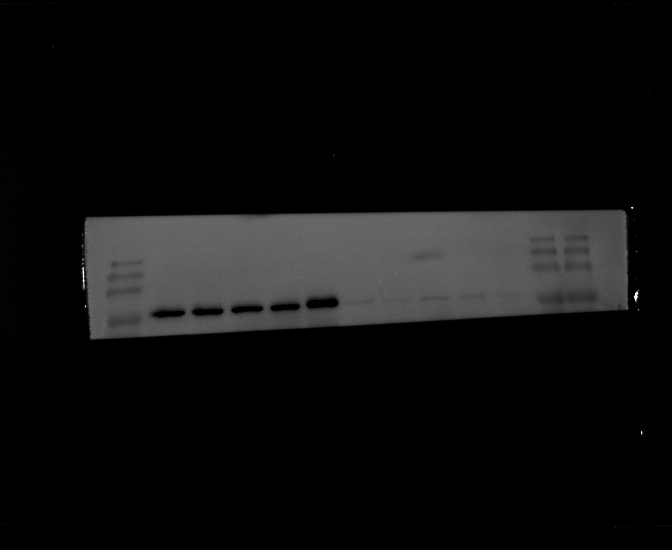

Supplement: Figure 6—source data 2. [file elife-96798-fig6-data2.zip › Figure6-source data2/RIP1_marker.tif]

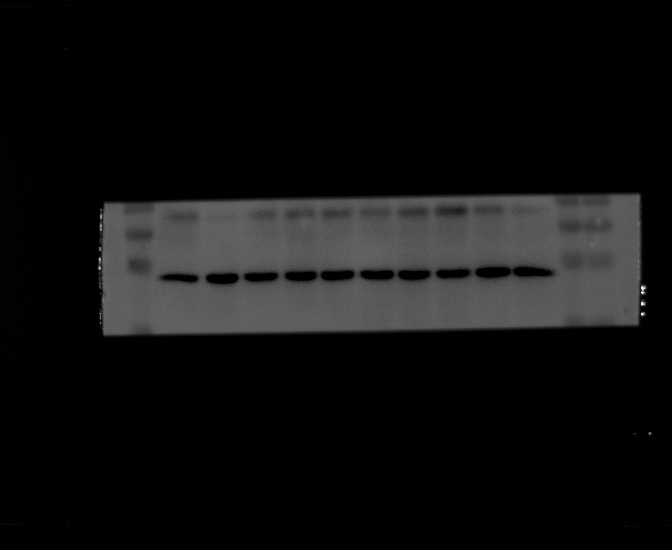

Supplement: Figure 6—source data 2. [file elife-96798-fig6-data2.zip › Figure6-source data2/GAPDH_marker.tif]

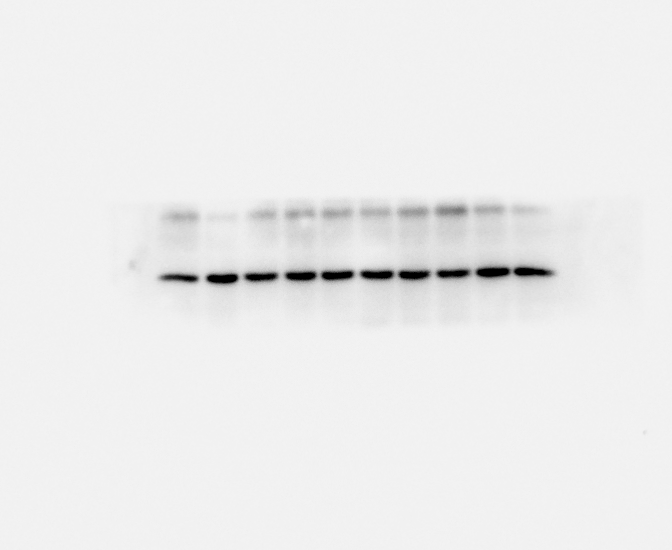

Supplement: Figure 6—source data 2. [file elife-96798-fig6-data2.zip › Figure6-source data2/GAPDH_sample.tif]

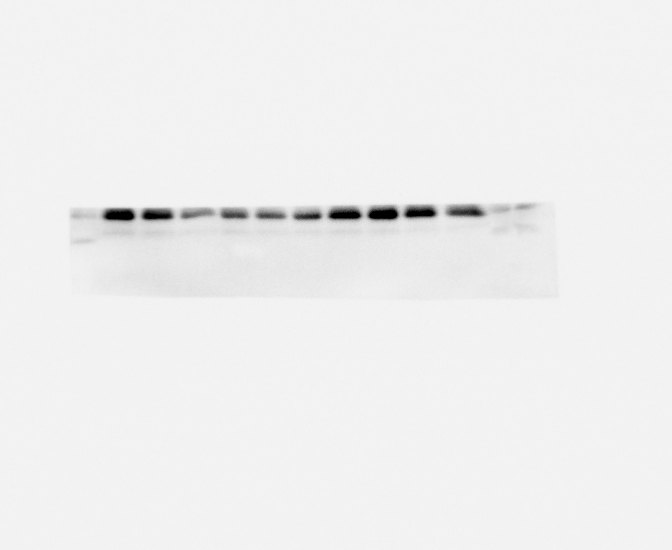

Supplement: Figure 6—source data 2. [file elife-96798-fig6-data2.zip › Figure6-source data2/TNFa_sample.tif]

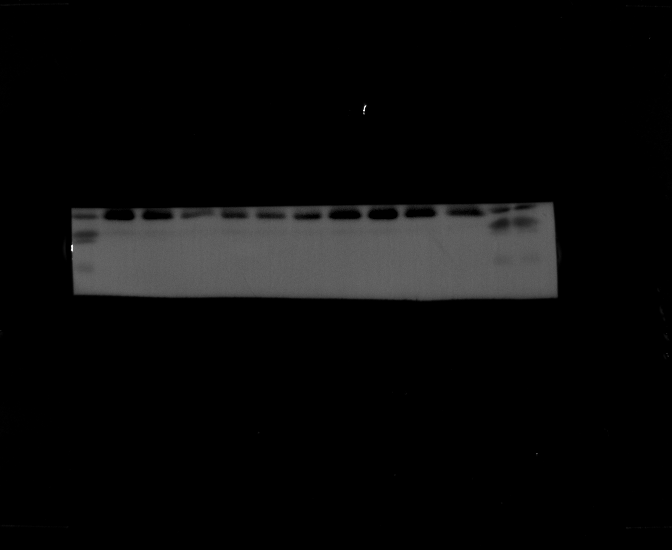

Supplement: Figure 6—source data 2. [file elife-96798-fig6-data2.zip › Figure6-source data2/TNFa_marker.tif]

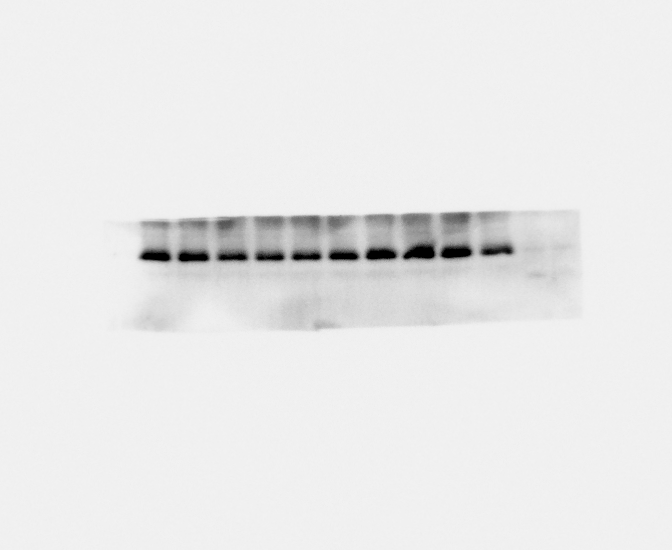

Supplement: Figure 6—source data 2. [file elife-96798-fig6-data2.zip › Figure6-source data2/CHOP_sample.tif]

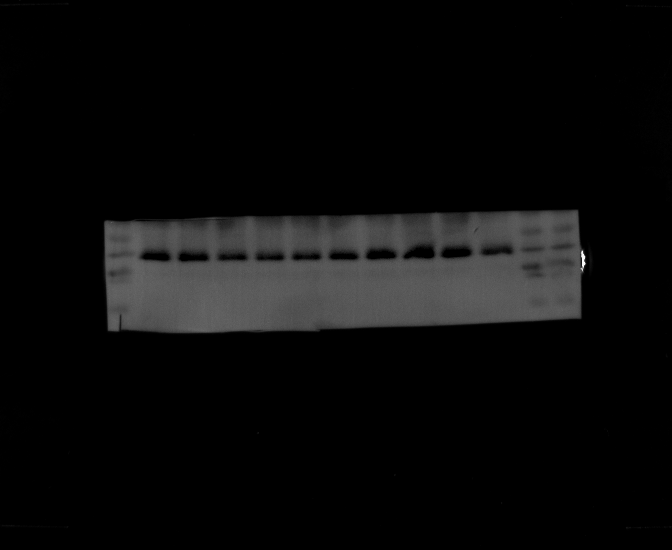

Supplement: Figure 6—source data 2. [file elife-96798-fig6-data2.zip › Figure6-source data2/CHOP_marker.tif]

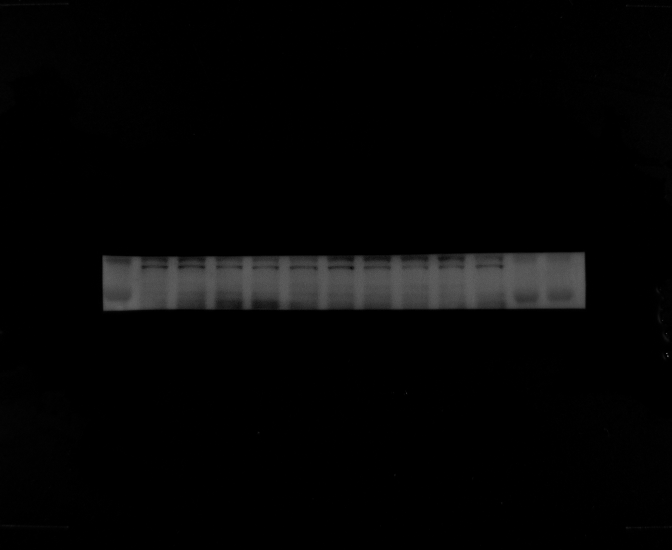

Supplement: Figure 6—source data 2. [file elife-96798-fig6-data2.zip › Figure6-source data2/GRP78_marker.tif]

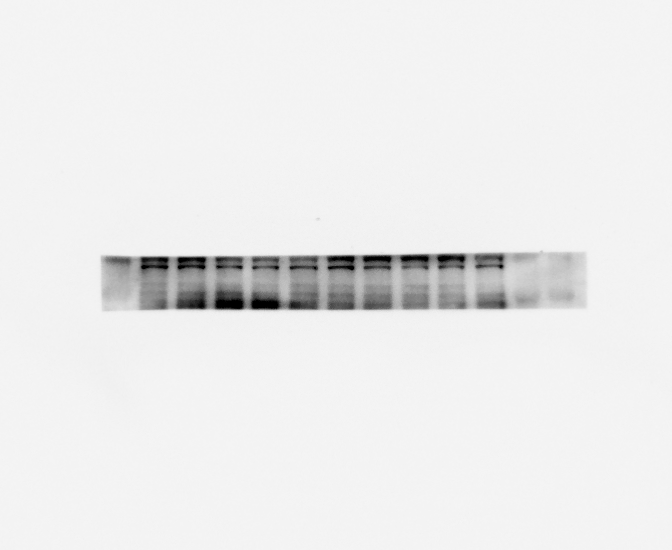

Supplement: Figure 6—source data 2. [file elife-96798-fig6-data2.zip › Figure6-source data2/GRP78_sample.tif]

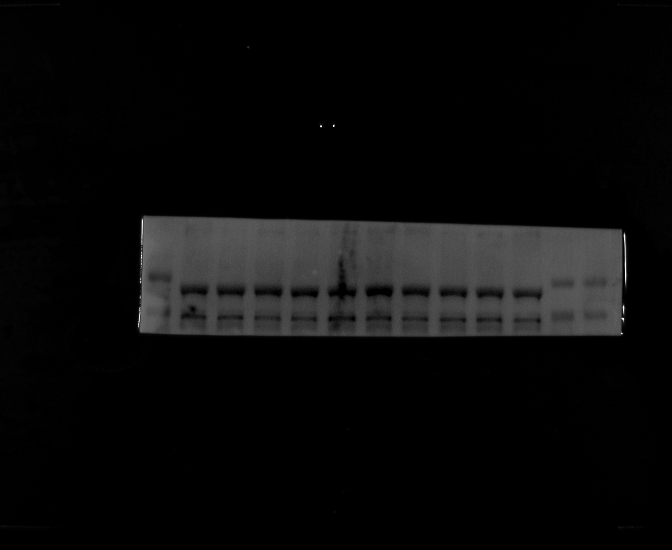

Supplement: Figure 6—source data 2. [file elife-96798-fig6-data2.zip › Figure6-source data2/IRE1a-4_marker.tif]

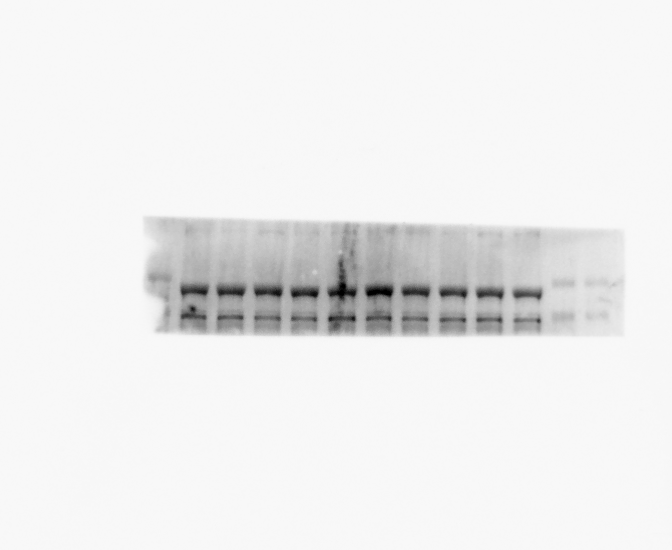

Supplement: Figure 6—source data 2. [file elife-96798-fig6-data2.zip › Figure6-source data2/IRE1a-4_sample.tif]

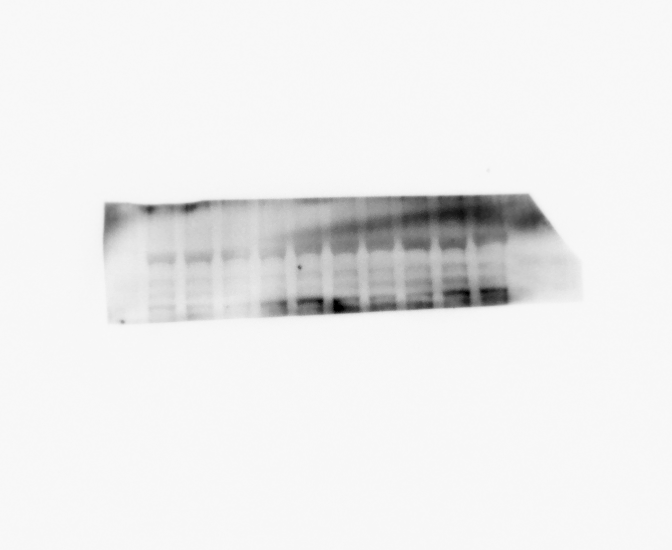

Supplement: Figure 6—source data 2. [file elife-96798-fig6-data2.zip › Figure6-source data2/p-ire1a_sample.tif]

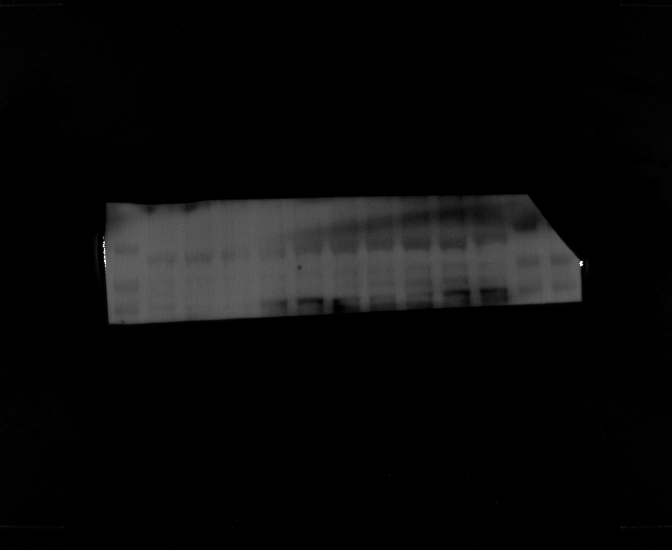

Supplement: Figure 6—source data 2. [file elife-96798-fig6-data2.zip › Figure6-source data2/p-ire1a_marker.tif]
